# Prediction of Functional Outcome After Acute Ischemic Stroke: Comparison of the CT-DRAGON Score and a Reduced Features Set

**DOI:** 10.3389/fneur.2020.00718

**Published:** 2020-07-31

**Authors:** Anouk Lesenne, Jef Grieten, Ludovic Ernon, Alain Wibail, Luc Stockx, Patrick F. Wouters, Leentje Dreesen, Elly Vandermeulen, Sam Van Boxstael, Pascal Vanelderen, Sven Van Poucke, Joris Vundelinckx, Sofie Van Cauter, Dieter Mesotten

**Affiliations:** ^1^Department of Anesthesiology and Perioperative Medicine, Ghent University Hospital, Ghent, Belgium; ^2^Department of Critical Care Services, Ziekenhuis Oost-Limburg Genk, Genk, Belgium; ^3^Department of Anesthesiology, VU University Amsterdam, Amsterdam, Netherlands; ^4^Department of Neurology, Ziekenhuis Oost-Limburg Genk, Genk, Belgium; ^5^Department of Medical Imaging, Ziekenhuis Oost-Limburg Genk, Genk, Belgium; ^6^UHasselt, Faculty of Medicine and Life Sciences, Diepenbeek, Belgium

**Keywords:** cerebrovascular disorders, stroke, prognosis, machine learning, thrombectomy, thrombolytic therapy, ischemic stroke, transient ischemic attack (TIA), mortality/survival, quality and outcomes, revascularization, treatment

## Abstract

**Background and Purpose:** The CT-DRAGON score was developed to predict long-term functional outcome after acute stroke in the anterior circulation treated by thrombolysis. Its implementation in clinical practice may be hampered by its plethora of variables. The current study was designed to develop and evaluate an alternative score, as a reduced set of features, derived from the original CT-DRAGON score.

**Methods:** This single-center retrospective study included 564 patients treated for stroke, in the anterior and the posterior circulation. At 90 days, favorable [modified Rankin Scale score (mRS) of 0–2] and miserable outcome (mRS of 5–6) were predicted by the CT-DRAGON in 427 patients. Bootstrap forests selected the most relevant parameters of the CT-DRAGON, in order to develop a reduced set of features. Discrimination, calibration and misclassification of both models were tested.

**Results:** The area under the receiver operating characteristic curve (AUROC) for the CT-DRAGON was 0.78 (95% CI 0.74–0.81) for favorable and 0.78 (95% CI 0.72-0.83) for miserable outcome. Misclassification was 29% for favorable and 13.5% for miserable outcome, with a 100% specificity for the latter. National Institutes of Health Stroke Scale (NIHSS), pre-stroke mRS and age were identified as the strongest contributors to favorable and miserable outcome and named the reduced features set. While CT-DRAGON was only available in 323 patients (57%), the reduced features set could be calculated in 515 patients (91%) (*p* < 0.001). Misclassification was 25.8% for favorable and 14.4% for miserable outcome, with a 97% specificity for miserable outcome. The reduced features set had better discriminative power than CT-DRAGON for both outcomes (both *p* < 0.005), with an AUROC of 0.82 (95% CI 0.79–0.86) and 0.83 (95% CI 0.77–0.87) for favorable and miserable outcome, respectively.

**Conclusions:** The CT-DRAGON score revealed acceptable discrimination in our cohort of both anterior and posterior circulation strokes, receiving all treatment modalities. The reduced features set could be measured in a larger cohort and with better discrimination. However, the reduced features set needs further validation in a prospective, multicentre study.

**Clinical Trial Registration**: http://www.clinicaltrials.gov. Identifiers: NCT03355690, NCT04092543.

## Introduction

With an incidence of 14 million patients annually, ischemic stroke is the second largest cause of death globally after ischemic heart disease. It has a major burden of morbidity as well, with an estimated annual 52 million disability-adjusted life years (1). Prognostic tools that predict outcome of acute ischemic stroke potentially provide early identification of patients who are likely to have a good or poor outcome despite treatment. If this tool has a high specificity for miserable outcome, it could be helpful in counseling patients and relatives, because estimations of outcome in stroke patients remain largely subjective at this moment. Moreover, and probably more applicable, these scores could be used for case-mix adjustments for benchmarking purposes.

In this view, several prognostic scoring systems have been developed to address this need, such as the ASTRAL, the CT-DRAGON, the iSCORE and the PLAN score (2). However, they have not been widely implemented in clinical practice, due to several limitations. First, a large number of input variables is required, some of which are hard to determine in the acute setting. Second, these scores are often tailored to subpopulations of stroke patients, depending on the localization of the stroke and/or the treatment received. Third, there is still a lack of validation in large patient populations using real world data, which are more prone to missingness and inaccuracies.

Treatment options for acute stroke have strongly evolved over the last decade. Most scoring systems were developed in the era of thrombolysis, while thrombectomy and its combination with thrombolysis only have been implemented over the last years (3–9). These developments certainly should have influenced reliable estimates of outcome and long-term effect of treatment.

To enhance implementation of prognostic tools, Fahey et al. advised to validate existing prognostic tools in different patient populations and treatment settings, opposed to designing new ones (10). Notably the CT-DRAGON score (Dense Artery, modified Rankin Scale, Age, Glucose, Onset-to-Treatment and NIHSS) has already been validated in previous studies and adapted to different diagnostics and treatments in stroke patients (11–17). The MRI-DRAGON score was developed and externally validated to deal with patients, in whom MRI was used as the first-line diagnostic tool (18, 19). Recently, the DRAGON score has also been modified to deal with patients, undergoing mechanical thrombectomy (20). These modifications are relevant since decision to perform mechanical thrombectomy in patients with wake-up strokes is often based on MRI diffusion-weighted imaging (DWI). Despite the modifications to specific subpopulations of stroke patients, its potential remains underutilized because of the missing data. A “light version” of the DRAGON score with less input variables, selected by machine learning, might thus be an alternative.

Machine learning has many applications, among which prediction of outcomes in healthcare. Machine learning techniques consist of algorithms able to solve problems by learning from experiences. Mathematical models are built and trained by providing training data. When new data are supplied, the models are able to generalize their learned expertise and make accurate predictions. Dimensionality reduction is a more recent application of machine learning. This process strives to reduce the number of variables under consideration. By “feature selection” input variables from existing scoring systems can be reduced and optimized (21, 22). With a reduced features set dynamic predictive models can be built, deployed and monitored over time.

We aimed to validate the CT-DRAGON score in all ischemic stroke localizations and for all treatment options, including a conservative treatment policy. The predictive power was then compared with a model, that included a set of the individual components of the CT-DRAGON score, selected by machine learning techniques.

## Methods

### Patient Population

This retrospective observational study included patients admitted to the stroke center of Ziekenhuis Oost-Limburg Genk, Genk, Belgium from January 2017 until February 2019. Patients with hemorrhagic stroke and stroke mimics were excluded. The final cohort comprised not only patients with acute ischemic stroke independent of pre-stroke mRS, stroke localization or treatment, but also patients with transient ischaemic attack (TIA) with symptoms on admission. These patients with TIA were included in the analysis, since its final diagnosis is often impossible to establish in an acute time frame. Treatment of stroke patients was categorized into (1) conservative therapy, (2) thrombolysis, (3) thrombectomy, or (4) a combination of thrombolysis and thrombectomy. This study was approved by the institutional review board of Ziekenhuis Oost-Limburg, Genk (19/0059U) and registered on ClinicalTrials.gov (NCT03355690, NCT04092543).

### Outcome Measure

Functional outcome was determined by the mRS score at 3 months. Patients were contacted by telephone within a timeframe of 3 months ± 2 weeks by the independent stroke study collaborator (EV) who was not involved in the care of the patients. Favorable outcome was defined as a mRS of 0, 1, or 2, miserable outcome as a mRS of 5 or 6 (11).

### Prediction Models

Data necessary for the calculation of the CT-DRAGON score were collected through a search of the electronic patient files. CT scans were evaluated by dedicated radiologists for the presence of a large vessel occlusion and early signs of infarction. A thrombus in the internal carotid artery or in the M1 or M2 segment of the middle cerebral artery on CT angiography was included in the score and was regarded as an alternative to the hyperdense cerebral artery sign (1 point). For strokes in the posterior circulation, the same principle was applied for an occlusion in the vertebral or basilar artery (1 point).

For ischemic strokes in the vascular territory of the medial cerebral artery, early infarct signs were scored (1 point) when the Alberta Stroke Program Early CT score (ASPECTS) was < 10. For all other vascular territories, any signs of early ischemia on CT scored 1 point. Early infarct signs on CT were defined as hypodense areas in any cerebral artery territory in combination with effacement of the adjacent sulci and loss of differentiation between gray and white matter.

As the sample size of the dataset was fixed, it was decided that the ratio of events (favorable or miserable outcome) per each candidate predictive factor should be at least 10.

While “time onset stroke to treatment” was one of the parameters in the original CT-DRAGON score, we used “time onset stroke to emergency department admission” as we did not want to exclude patients who received conservative treatment. When the time of onset was unknown, it was assumed to be more than 90 min and thus scoring 1 point. In this way a complete-case analysis was employed, with missing data from the other parameters of the CT-DRAGON recoded as 0 points. While this rigid, arbitrary imputation of missing values is clinically intuitive, it may inherently induce bias as the data are most likely not missing at random.

Through machine learning techniques, we selected the parameters of the CT-DRAGON score that did or did not provide additional predictive value. By use of logistic regression, decision tree analysis (maximum of 4 splits) and bootstrap forest in sequential order a reduced features set with the most powerful predictors of the CT-DRAGON was built. The 7 components of the CT-DRAGON score were added into the models as a continuum over the entire range of the values (age in years, NIHSS in ordinal points, pre-stroke mRS in ordinal points, glycaemia in mg/dL, time to emergency department in hours). Early ischaemia (yes/no) and dense artery sign or its posterior equivalent (yes/no) were entered as dichotomous values. The three models were only used to assess the consistency of the prognostic factors in terms of selection. The ranking of factors was done by bootstrap forests to generate the contributions. This process was run three times and the average was taken. The number of elements for the reduced features set was not predefined. Instead, the cumulative relative contribution to the outcome had to be at least 80%, when combining the factors with the highest relative contribution. The bootstrap forest analysis fits an ensemble model by averaging many decision trees each of which is fit to a bootstrap sample of the training data. Each split in each tree considers a random subset of the predictors. In this way, many weak models are combined to produce a more powerful model. A minimum of 10 splits per tree and a minimum size of 5 per tree was used.

We then tested the reduced features set in a training and a validation cohort. Baseline patient characteristics were compared between training and validation cohorts. Finally, the predictive performance of the CT-DRAGON and the reduced features set were compared for discrimination, calibration and misclassification. For these analyses patients, in whom the mRS at 90 days was missing, were excluded.

### Statistical Analyses

The distribution of data was analyzed. All data were represented as either mean +/− SD or median and IQR. A training (0.75 of study cohort) and a validation cohort (0.25) were established by random selection. We assessed the performance of the CT-DRAGON and the reduced features set with the variance and the AUROC for both favorable and miserable outcomes. Calibration of the model was tested by lack of fit. Misclassification rate, sensitivity and specificity were calculated. We estimated the fit of the model with the Akaike Information criterion (AIC). Analyses were performed in JMP Pro, version 14.1.0 (SAS Institute Inc, Cary, NC, USA). Two-sided *P* < 0.05 were deemed statistically significant.

## Results

### Descriptive Statistics

**Figure 1:**
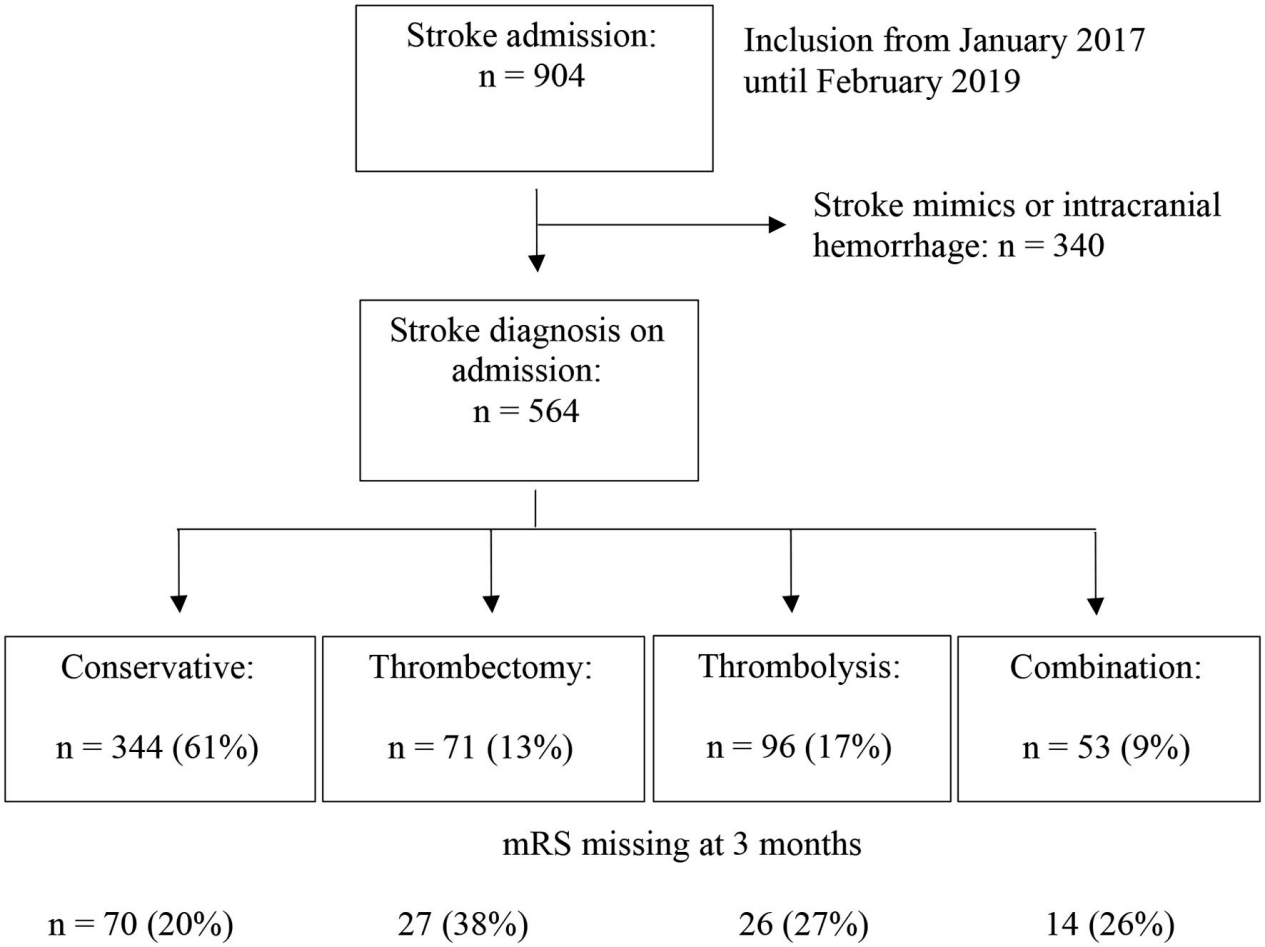
Flowchart of patients.

From January 2017 to February 2019 a total of 904 patients with stroke symptoms were admitted (Figure 1). Of these, 564 were diagnosed with ischemic stroke or TIA with symptoms on admission. The mean age was 73 years (SD 13) and 296 (52%) patients were male (Table 1). The median NIHSS on admission was 6 (IQR 3–14). Three hundred and forty-four (61%) patients received conservative treatment. This represents a heterogeneous group of patients with on the one hand no indication for thrombolysis or thrombectomy due to the low severity of the stroke and on the other hand patients with severe symptoms and contraindications for treatment. Ninety-six (17%) patients received thrombolysis, 71 (13%) patients underwent thrombectomy and 53 (9%) patients were treated with the combination of thrombolysis and thrombectomy.

**Table 1:** Baseline patient characteristics.

***N* = 564**		**Missings, *n***
Age, years, mean (SD)	73 (13)	
Sex, *n* (%)		
Male	296 (52)	
National Institutes of Health Stroke Scale (NIHSS), median (IQR)	6 (3–14)	29
Glycemia, mg/dL, mean (SD)	128 (44)	46
Time onset stroke to emergency department (ED) admission, hours, median (IQR)	1.7 (0.9–3.8)	184
Localization, *n* (%)		
Anterior	182 (32)	
Posterior proximally	12 (2)	
Lacunar	280 (50)	
No large vessel occlusion	90 (16)	
Pre-stroke modified Rankin Scale score (mRS), *n* (%)		39
0	237 (45)	
1	158 (30)	
2	49 (9)	
3	54 (10)	
4	19 (4)	
5	8 (2)	
Hyperdense artery/vessel occlusion, *n* (%)	194 (34)	
Alberta Stroke Program Early CT score (ASPECTS), *n* (%)		
<10	149 (34)	
Early infarct signs, *n* (%)		
Yes	39 (35)	
ASPECTS + early infarct signs, *n*		16
CT-DRAGON score, mean (SD)	4 (2)	241
Treatment, *n* (%)		
Conservative treatment	344 (61)	
Thrombectomy	71 (13)	
Thrombolysis	96 (17)	
Thrombectomy + thrombolysis	53 (9)	

One hundred eighty-two (32%) patients suffered a stroke in the anterior cerebral circulation. Twelve (2%) patients had a proximal posterior ischemic stroke, 280 (50%) had a lacunar infarction and in 90 (16%) patients no large vessel occlusion could be visualized. The validation cohort did not differ from the training cohort for baseline characteristics (Appendix D).

The mean CT-DRAGON score for the whole population was 4 ± 2. The mean CT-DRAGON scores, subdivided by treatment group, are represented in Table 2. The scores were different between the thrombectomy (± thrombolysis) and the thrombolysis or the conservative treatment group (*p* < 0.0001).

**Table 2:** Patient characteristics by treatment.

***N* = 564**	**Conservative (*n* = 344)**	**Thrombectomy (*n* = 71)**	**Thrombolysis (*n* = 96)**	**Combination (*n* = 53)**
Age years, mean (SD)	74 (13)	74 (14)	72 (14)	75 (13)
Sex, *n* (%)				
Male	188 (55)	33 (46)	50 (52)	25 (47)
NIHSS, median (IQR)	4 (2–8)	17 (14–22)	6 (4–12)	16 (10–20)
Minor stroke (NIHSS <4), *n* (%)	151 (45)	5 (8)	22 (24)	2 (4)
Glycemia, mg/dL, mean (SD)	128 (46)	136 (40)	123 (45)	125 (38)
Time onset stroke to ED, hours, median (IQR)	2.5 (1.1–5)	1.7 (0.8–3.2)	1.2 (0.7–2,1)	1.3 (0.8–3.3)
Localization, *n* (%)				
Anterior	50 (15)	63 (89)	32 (33)	48 (91)
Posterior proximally	2 (1)	6 (8)	2 (2)	3 (5)
Lacunar	222 (64)	2 (3)	50 (52)	2 (4)
No large vessel occlusion	70 (20)	0	12 (13)	0
Pre-stroke mRS, median (IQR)	1 (0–2)	1 (0–2)	1 (0–1)	0 (0–1)
ASPECTS, *n* (%)				
<10	81 (31)	25 (48)	16 (20)	27 (60)
10	179 (69)	27 (52)	65 (80)	18 (40)
Early infarct signs, *n* (%)				
Yes	28 (36)	6 (50)	1 (7)	4 (80)
No	50 (64)	6 (50)	14 (93)	1 (20)
CT-DRAGON, mean (SD)	3 (2)	6 (2)	3 (2)	6 (2)

While mRS at 3 months was missing in 137 (24%) cases, mortality (16%, mRS 6) was available for all patients (Table 3). Two hundred and forty-six (58%) patients had a favorable outcome (mRS 0–2). Eighty-four (19%) patients had a miserable outcome (mRS 5–6).

**Table 3:** Modified rankin scale score at 90 days.

***N* = 427**		
**Missings:** ***n*** **= 137**	***N***	**Proportion**
0	97	23%
1	104	24%
2	45	11%
3	44	10%
4	53	12%
5	14	3%
6	70	16%

### Development of the Proposed Reduced Features Set

Logistic regression, bootstrap forest and decision tree analysis showed comparable performance of the individual components in determining both favorable and miserable outcome. Age, NIHSS and pre-stroke mRS were the most predictive parameters from the CT-DRAGON score in bootstrap forest analysis, with a proportional contribution to the outcome of 87% for the prediction of favorable outcome and 85% for the prediction of miserable outcome (Table 4). The reduced features set could be calculated in 515 patients (91%) in comparison with the CT-DRAGON score, which could only be determined in 323 patients (57%) (*p* < 0.001).

**Table 4:** Feature selection by bootstrap forest for favorable and miserable outcome.

**Predictor**	**Contribution**	**Proportion**	**Rank**
**FAVORABLE OUTCOME VS. OTHER**			
NIHSS	20.21	0.40	1
Pre-stroke mRS	13.78	0.27	2
Age	10.14	0.20	3
Glycemia	3.99	0.08	4
Time onset to ED admission	1.81	0.03	5
Early infarct signs	1.13	0.02	6
**MISERABLE OUTCOME VS. OTHER**			
NIHSS	14.76	0.43	1
Pre-stroke mRS	9.12	0.27	2
Age	5.03	0.15	3
Glycemia	2.78	0.08	4
Time onset to ED admission	2.18	0.06	5
Early infarct signs	0.40	0.01	6

### Analytical Statistics

The AUROC for the CT-DRAGON score in predicting favorable outcome was 0.78 (95% CI 0.74–0.81) (Table 5). For predicting miserable outcome, it was 0.78 (95% CI 0.72–0.83). The misclassification rate of the CT-DRAGON in predicting favorable and miserable outcome was 29 and 13.5%, respectively. Sensitivity and specificity were 67 and 74% in the prediction of favorable and 10 and 100% in the prediction of miserable outcome. *R*^2^ was 0.30 (lack of fit *p* = 0.91) and 0.22 (lack of fit *p* = 0.76) for the prediction of favorable and miserable outcome, respectively, by the CT-DRAGON score.

**Table 5:** Area under the receiver operating characteristic curve (AUROC), misclassification rate, sensitivity, specificity, variance, and Akaike Information Criterion (AIC) of the CT-DRAGON and the reduced features set.

	**Favorable outcome vs. other**	**Miserable outcome vs. other**
CT-DRAGON score	AUROC 0.78 (95% CI 0.74–0.81) Misclassification rate 29% Sensitivity 67% Specificity 74% *R*^2^ 0.30 AIC 632	AUROC 0.78 (95% CI 0.72–0.83) Misclassification rate 13.5% Sensitivity 10% Specificity 100% *R*^2^ 0.22 AIC 397
Reduced features set	AUROC 0.82 (95% CI 0.79–0.86) Misclassification rate 25.8% Sensitivity 77% Specificity 73% *R*^2^ 0.42 AIC 528	AUROC 0.83 (95% CI 0.77–0.87) Misclassification rate 14.4% Sensitivity 23% Specificity 97% *R*^2^ 0.29 AIC 346

We found an AUROC of 0.82 (95% CI 0.79–0.86) and 0.83 (95% CI 0.77–0.87) in predicting favorable and miserable outcome, respectively, based on age, NIHSS and pre-stroke mRS (the reduced features set). Misclassification rates were 25.8% for favorable and 14.4% for miserable outcome. Sensitivity and specificity were 77 and 73%, respectively, in the prediction of favorable and 23 and 97%, respectively, in the prediction of miserable outcome. *R*^2^ was 0.42 (lack of fit *p* = 0.34) and 0.29 (lack of fit *p* = 1.0) for the prediction of favorable and miserable outcome, respectively, by the reduced features set. However, the discrimination of the reduced features set was lower in the patient population in which the CT-DRAGON could be calculated (*n* = 323) with an AUROC of 0.78 for favorable and 0.79 for miserable outcome.

For the prediction of favorable and miserable outcome, the AIC was 632 and 397, respectively, based on the CT-DRAGON and 528 and 346, respectively, based on the reduced features set.

## Discussion

This study could confirm that the CT-DRAGON score is a useful and validated tool to predict long-term functional outcome after acute ischemic stroke. Yet the collection of all necessary input data for this score seems challenging in a real-world setting. Dimensionality reduction of the rather complex CT-DRAGON score yielded a reduced features set with similar predictive characteristics. Its input parameters (age, NIHSS and pre-stroke mRS) can be rapidly obtained by history taking and clinical neurological examination, leading to a much broader implementation potential. It hence does not depend on radiological findings, which require a specialist's evaluation.

The original CT-DRAGON score and the reduced features set were validated together in a well-controlled single-center dataset of patients, who suffered from anterior or posterior circulation or lacunar strokes, treated with all modalities, including basic antiplatelet therapy. It has already been shown that discrimination does not differ between anterior and posterior strokes in a large cohort of patients, who received intravenous thrombolysis, with a AUROC of approximately 0.83 (12). A small study validated the DRAGON score in patients treated with the combination of thrombolysis and thrombectomy (14). However, neither the AUROC was calculated, nor a development and validation cohort were used. The predictive performance of the CT-DRAGON in the large multicentre Safe Implementation of Thrombolysis in Stroke-International Stroke Thrombolysis Register (SITS-ISTR) showed a similar specificity of 74.1–99.7% for the prediction of good and poor outcome (13). The CT-DRAGON score also had a better discrimination for miserable outcome (AUROC 0.89) than for good outcome (AUROC 0.79) in a Danish cohort of patients undergoing IV thrombolysis (15). The predictive performance (AUROC 0.84) of the CT-DRAGON score in a Spanish cohort with more severe strokes was comparable to the latter external validation studies (16). The discrimination of the CT-DRAGON for good (AUROC 0.73) and miserable (AUROC 0.75) outcome was lower compared to the previous studies and more in line with our study (17). The lower predictive performance is partially explained by a lower number of patients with high CT-DRAGON scores (>7). Our study also included many patients with relatively low CT-DRAGON scores, with only 10% of the patients having a CT-DRAGON score of >7. Taking together the entire body of external validation studies, the CT-DRAGON score can be implemented for the prognostication of functional outcome after acute ischemic stroke. However, the score seems to perform better in patient with more severe strokes.

Whether the CT-DRAGON score has a better discrimination in patients undergoing thrombectomy, whether or not in combination with thrombolysis, could not be determined in our study, as the number of these patients was too low for subgroup analyses. It can be assumed that the AUROC will at least be in line with the one of the patients undergoing thrombolysis for strokes with higher severity. The study by Ovesen showed that in patients with a M1 occlusion, a typical indication for thrombectomy, the AUROC of the CT-DRAGON score for miserable outcome was 0.89 (15).

Despite these merits, the CT-DRAGON is not being applied routinely in daily practice. The lack of availability of some variables at admission and the number of parameters is a potential limitation for clinicians in a usually busy emergency department. This is reflected in the large number of missing values in our data, which excludes patients and their relatives to have benefit from prognostication and which may introduce significant bias into outcome data. Missing values are rarely random in a clinical context and multiple data imputation to account for missing values, is not a practical solution in daily clinical practice. Practical assumptions on missing data, as we used in our study, may on the other hand lead to underestimation of the CT-DRAGON score, undermining its potential.

We therefore used the principle of dimensionality reduction to select the features from the CT-DRAGON score which contribute the most to the predictive power of the original score. The selection of the most predictive parameters—NIHSS, age and pre-stroke modified Rankin Scale—was done by bootstrap forest. In this machine learning process, missingness is also taken into account. This is a confirmation of the development of the original CT-DRAGON score by logistic regression (11). Here the variables associated with the strongest regression coefficients were also NIHSS, age and pre-stroke mRS. It indicates that the strongest predictors for functional outcome remained stable, even after the introduction of thrombectomy and the inclusion of patients with posterior strokes in the study population, and it underpins the validity of the core of the CT-DRAGON score.

This reduced features set could be scored in a larger proportion of the stroke population, increasing its generalizability and applicability in patients suffering from posterior circulation and lacunar strokes as well. Additionally, the reduced features set had a slightly better performance, compared to the CT-DRAGON, for the prediction of good and miserable outcome. Due to the limited parameters, the reduced features set is less vulnerable to overfitting, reflected by the lower AIC and BIC values. However, the better performance of the reduced features set may be biased as missing variables in the CT-DRAGON score were higher, hence impairing its performance. Most likely, the CT-DRAGON would have a better discrimination than the reduced features set, if all data for the score would always be available, which is unfortunately not the case in a real-world setting.

The “simple variables model,” using age, pre-stroke functional status, living alone pre-stroke, being able to walk unaided, lift both arms off the bed and have a normal verbal Glasgow Coma Score, has also been compared to the CT-DRAGON score (23). Here the simplified score had a comparable performance for predicting miserable outcome, compared with the CT-DRAGON score. However, its six variables are comparable to the number of variables of the CT-DRAGON score. In contrast, Wouters et al. obtained a similar AUROC for a model using only age and NIHSS in prediction of good outcome (0.82 vs. 0.78 in our study) (24). In their study the inclusion of the difference in NIHSS between admission and after 24 h did not add much predictive power. The variance of NIHSS in the prediction of outcome was 11–25% in the analysis by Rost et al. (25).

Both the original CT-DRAGON score and reduced features set were much better in predicting miserable outcome with a high specificity. Since none of the prognostic scales are yet incorporated in routine clinical practice, the combination of age, pre-stroke modified Rankin Scale and NIHSS as a reduced features set may therefore provide a good alternative. It is immediately available, easier to implement and has a better performance than the original CT-DRAGON, while maintaining an excellent specificity in predicting miserable outcome. However, dynamic predictive models require machine learning techniques to be built, deployed and monitored over time. This makes these dynamic models far less intuitive and practical than rigid scoring systems.

This study has several limitations. First, the monocentric design and the retrospective analysis of the prospectively collected data prevent to make definitive conclusions on the predictive performance of the reduced features set. Second, there was a major bias due to the absence of the outcome, mRS at 3 months, in about a quarter of the patients. Certainly, if the outcome assessment would have differed among patient subpopulations, this may have further amplified the bias. Third, the inclusion of the patients who did not receive either thrombolysis or thrombectomy may have affected the performance of both scoring systems, as the reasons for abstinence of therapy have varied strongly. However, the aim of this study was the validation of a “score” with the knowledge available during the admission to the emergency department. Therefore, the reduced features set should still be validated prospectively in a large multicentric study using real-world data. To do this the reduced features set needs to be either converted in an intuitive, clinically comprehensible score or validated as a dynamic predictive model, requiring the continuous use of machine learning techniques. Another drawback of the latter model is the lack of a formal, explicit definition and the absence of an intuitive, easily comprehensible attribution of points, which are the cornerstones of rigid scoring systems. Fourth, the difference in performance between the CT-DRAGON score and the reduced features set was attenuated in the analysis of the subgroup in which all data were available. This points to an inherent bias against the CT-DRAGON score, due to its more pronounced vulnerability to missing data.

Finally, prediction of outcome at population level, is not equal to its performance in an individual patient. Furthermore, prognostication at individual patient level with the aim of therapeutic decision making may be unrealistic and not applicable. Scores could be used for case-mix adjustments for benchmarking purposes, driven by clinicians, in order to monitor quality of care over time and to compare outcomes among different centers.

In conclusion, a reduced features set using the readily obtainable parameters age, pre-stroke functional status and stroke severity can reasonably predict functional outcome and is a promising tool for case-mix adjustments in the purpose of benchmarking.

## Data Availability Statement

The datasets generated for this study are available on request to the corresponding author.

## Ethics Statement

The studies involving human participants were reviewed and approved by Ziekenhuis Oost-Limburg, Secretariaat Comité Medische Ethiek Schiepse Bos 6, 3600 GENK ec.submission@zol.be. Written informed consent for participation was not required for this study in accordance with the national legislation and the institutional requirements.

## Author Contributions

AL participated in the design of the study and analysis plan, checked the database for accuracy and exported the data for statistical analysis, performed the statistical analyses, and drafted the manuscript. JG drafted the manuscript. LE supervised patient recruitment, participated in the design of the study, and revising the manuscript for important intellectual content. AW, LS, SVB, and PV supervised patient recruitment and revising the manuscript for important intellectual content. PW, SVP, and JV revising the manuscript for important intellectual content. LD checked the database for accuracy and revising the manuscript for important intellectual content. EV follow-up of patients, checked the database for accuracy, and revising the manuscript for important intellectual content. SVC participated in the design of the study and drafted the manuscript. DM conceived the study, study design and analysis plan, performed the statistical analyses, and drafted the manuscript. All authors read and approved the final manuscript.

## Conflict of Interest

The authors declare that the research was conducted in the absence of any commercial or financial relationships that could be construed as a potential conflict of interest.
