# Supplementary material for: Prediction of Functional Outcome After Acute Ischemic Stroke: Comparison of the CT-DRAGON Score and a Reduced Features Set
Source: Front Neurol. 2020 Jul 31;11:718. doi: 10.3389/fneur.2020.00718 (PMC7412791; doi:10.3389/fneur.2020.00718)
Supplement: Supplementary file 1 [file Image_1.pdf]

APPENDIX

A. Original CT-DRAGON score versus CT-DRAGON score in the study

Original DRAGON score	CT-DRAGON score in the study
(Hyper)dense cerebral artery sign on admission CT head scan: no or yes (0 or 1 point)	Thrombus of the internal carotid, middle cerebral (M1 or M2), basilar or vertebral artery on admission CT head scan: no or yes (0 or 1 point)
Early infarct signs on admission CT head scan: no or yes (0 or 1 point)	ASPECTS <10 or early infarct signs on admission CT head scan: no or yes (0 or 1 point)
Pre-stroke modified Rankin Scale score >1: no or yes (0 or 1 point)	=
Age: <65, 65-79 or >79 years (0, 1 or 2 points)	=
Glucose level on admission: ≤8 mmol/L or >8 mmol/L (0 or 1 point)	=
Time onset stroke to treatment: ≤90 or >90 minutes (0 or 1 point)	Time onset stroke to emergency department admission: ≤90 or >90 minutes (0 or 1 point)
NIHSS on admission: 0-4, 5-9, 10-15 or >15 (0, 1, 2 or 3 points)	=

B. Definition of localisations

Localisation	Imaging
Anterior	• Thrombus in the internal carotid artery or<br>• Thrombus in the middle cerebral artery (M1 or M2)
Posterior proximally	• Thrombus in the basilar artery or<br>• Thrombus in the vertebral artery
Lacunar	Infarction with absence of thrombus in the internal carotid, middle cerebral (M1 or M2), basilar or vertebral artery.
No large vessel occlusion	No infarction

C. Recoding to obtain complete case group

Parameters of the CT-DRAGON score	Recode missings as
ASPECTS/early infarct signs on admission CT head scan	0
Pre-stroke modified Rankin Scale score	0
Glucose level on admission	0
Time onset stroke to emergency department admission	1
NIHSS on admission	0

D. Patient baseline characteristics of the training versus the validation cohort

N = 564	Training cohort	Validation cohort
Age years, mean (SD)	74 (13)	73 (14)
Sex, n (%)		
Male	228 (54)	68 (49)
NIHSS, median (IQR)	6 (3-15)	6 (3-14)
Glycemia, mg/dL, mean (SD)	129 (46)	126 (39)
Time onset stroke to ED admission, hours, median (IQR)	1.6 (0.9-3.8)	1.9 (0.8-3.8)
Pre-stroke mRS, median (IQR)		
>1, n (%)	98 (25)	32 (25)
ASPECTS, n (%)		
<10	108 (33)	41 (38)
10	223 (67)	66 (62)
Early infarct signs, n (%)		
Yes	29 (35)	10 (36)
No	53 (65)	18 (64)
